# Films based on crosslinked TEMPO-oxidized cellulose and predictive analysis via machine learning

**DOI:** 10.1038/s41598-018-23114-x

**Published:** 2018-03-16

**Authors:** Merve Özkan, Maryam Borghei, Alp Karakoç, Orlando J. Rojas, Jouni Paltakari

**Affiliations:** Department of Bioproducts and Biosystems, School of Chemical Technology, Aalto University, Espoo, Finland

## Abstract

We systematically investigated the effect of film-forming polyvinyl alcohol and crosslinkers, glyoxal and ammonium zirconium carbonate, on the optical and surface properties of films produced from TEMPO-oxidized cellulose nanofibers (TOCNFs). In this regard, UV-light transmittance, surface roughness and wetting behavior of the films were assessed. Optimization was carried out as a function of film composition following the “random forest” machine learning algorithm for regression analysis. As a result, the design of tailor-made TOCNF-based films can be achieved with reduced experimental expenditure. We envision this approach to be useful in facilitating adoption of TOCNF for the design of emerging flexible electronics, and related platforms.

## Introduction

The increased demands of the future green bioeconomy have triggered efforts to substitute petroleum-based products with environmentally friendly alternatives^1^. In this aspect, nanocellulose based materials have shown promise for various applications, mainly owing to the possibility of obtaining transparent, flexible and recyclable materials^2^. However, any development needs demonstration of competitive performance relative to that of conventional substrates based on polyethylene terephthalate (PET) or polyethylene naphthalate (PEN)^1,3^. In this regard, films made of TEMPO-oxidized cellulose nanofibers (TOCNF) have promise, given their optical transparency achieved by the nanofibrils that carry carboxylic groups. Compared to unmodified cellulose nanofibers, TOCNF is more negative and displays a more extensive degree of fibrillation. Moreover, post treatments have been applied to further improve the properties of TOCNF films, for example, by impregnation in aqueous MgCl_2_, CaCl_2_, AlCl_3_ and FeCl_3_ electrolyte solutions, which have been found to increase water resistance via ion-exchange. Interfibrillar crosslinking via Mg^2+^ and Al^3+^ ions has been noted to exhibit 80% transmittance (600 nm) of thin films (15 ± 1 µm)^4^. Another application, the treatment with reactive sizing, via alkylketene dimer, reduced the hydrophilicity of TOCNF films without compromising their transparency. For instance, compared to the initial water contact angle of unmodified films (WCA ~47°), the WCA of AKD-modified TOCNF films increased significantly (~94°)^5^.

In this work, a film-forming biopolymer, polyvinyl alcohol (PVA), was combined with TOCNF networks, offering an alternative to the conventional cellulosic films in terms of biodegradability and stability^6^. PVA is available in different forms, depending on the degree of hydrolysis or deacetylation and molar mass and it is used in diverse applications ranging from paper coatings^7^, textile sizing^8^, agriculture engineering^9^ to clinical research^10^. Recently, PVA was used as a reinforcing agent to produce water-stable films of TEMPO/NaClO_2_ oxidized cellulosic nanofibrils^11^ and chemical crosslinking was provided through esterification of COOH in TOCNF with hydroxyl groups of PVA.

**Figure 1:**
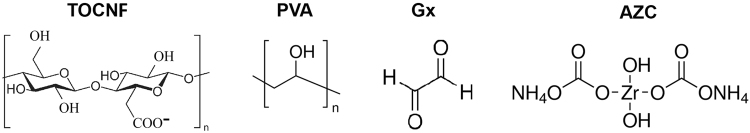
Basic chemical structure of TOCNF and PVA, Gx and AZC.

We investigate the influence of glyoxal (Gx) and ammonium zirconium carbonate (AZC), two common organic and inorganic crosslinkers, respectively, on the properties of TOCNF and composite TOCN-PVA films. Glyoxal is widely used in textile, paper, cosmetic, health and leather industries as viscosity modifier, strength enhancer for paper, softener for less wrinkled textiles or biocide^12^. Ammonium zirconium carbonate (AZC), on the other hand, interacts with the hydroxyl and carboxyl groups of the fibrils and creates electrostatic charges via protonation in water. AZC is safe for food-packaging applications^13^ and it is a crosslinker for PVA and cellulose in pulp and paper industries^14^. For instance, starch and cellulose, in the presence of AZC, form hydrogen bonding (fiber-starch, fiber-fiber, and starch-starch)^15^. The chemical structures of TEMPO-oxidized cellulose (TOCN), PVA, Gx and AZC are included in Fig. 1.

In this study, TOCNF, PVA, Gx and AZC were combined in different compositions to investigate their effects on the films in terms of surface and optical properties. Obtained data set was trained to predict other possible outcomes from generated samples by means of a machine learning algorithm. In particular, the random forest method was used given its power in data mining, identification, analysis and prediction. Especially, the method is popular among the researchers in the fields of finance, biology and chemistry due to its data handling efficiency and potential for capturing linear and non-linear interaction between input and output data^16^.

## Results

### Surface Analysis

**Figure 2:**
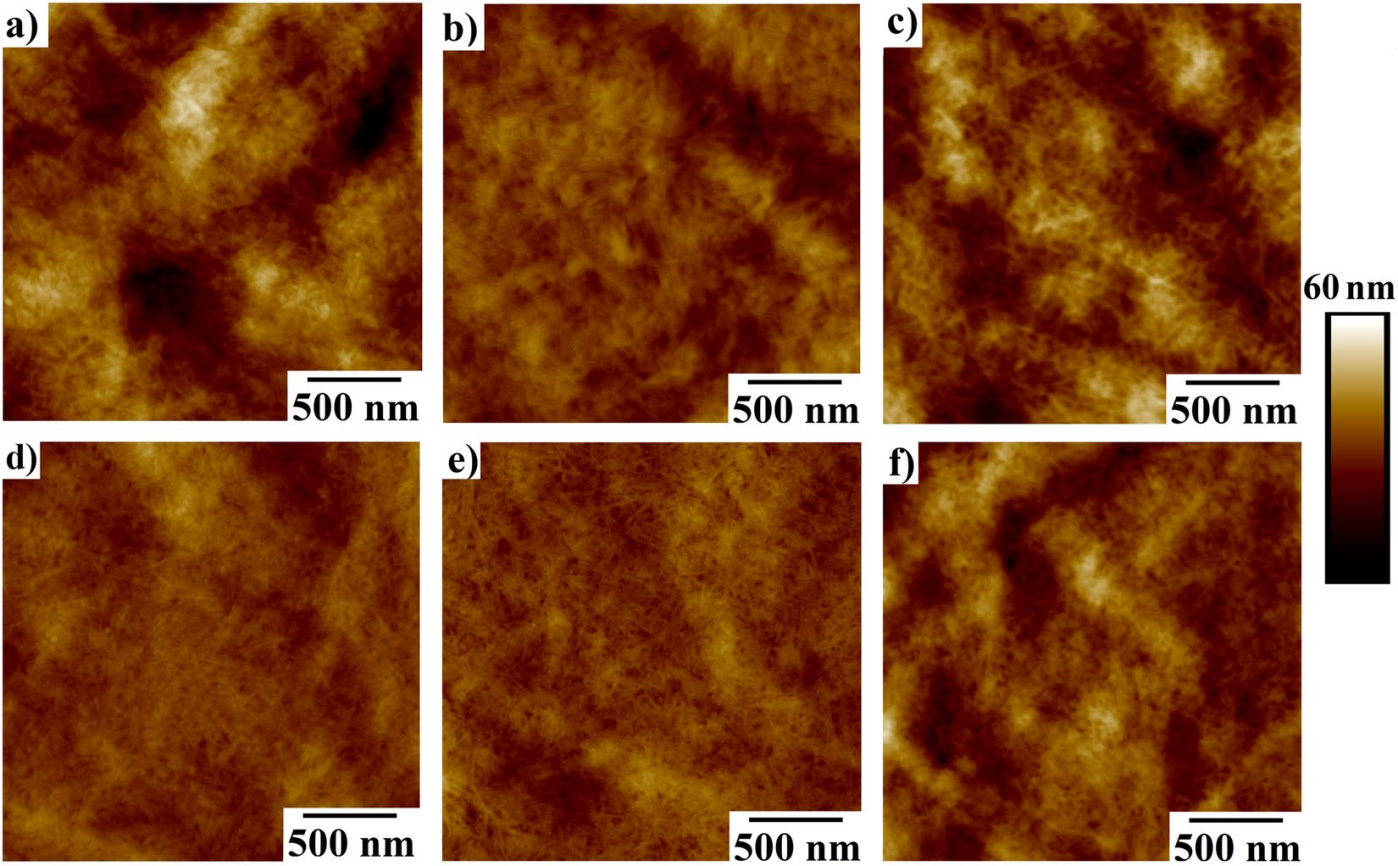
AFM images of (**a**) C, (**b**) CG10, (**c**) CA10, (**d**) CP10, **(e**) CP10G10 and (**f**) CP10A10 film surfaces.

Modification of the film surface opens to door to create new alternative substrates for various applications, such as, printed electronics^17^. Films consisting of different additives (PVA, Gx and AZC) and TOCNF were introduced in Methods chapter. Surface AFM images of C, CG10, CA10, CP10, CP10G10 and CP10A10 films are shown in Fig. 2a–f, respectively. Films prepared with TOCNF reveal a randomly distributed nanofibrillar structure (Fig. 2a)^5^. The incorporation of AZC and Gx resulted in a more homogenous surface topology, Fig. 2b,c. Likewise, introduction of PVA (CP10) resulted in a more uniform network. One reason might be that the cellulose fibrils form a tighter structure^18^. It can be inferred that addition of Gx to the CP10 enhanced distribution of fibrils leading to a more uniform surface, while incorporation of AZC into the CP10 caused more aggregation and uneven surfaces. This might be attributed to the smaller molecular size of Gx compared to AZC, with higher ratio of carboxylic group to the total weight resulting into stronger chemical bonds with the hydroxyl groups of PVA and cellulose.

**Figure 3:**
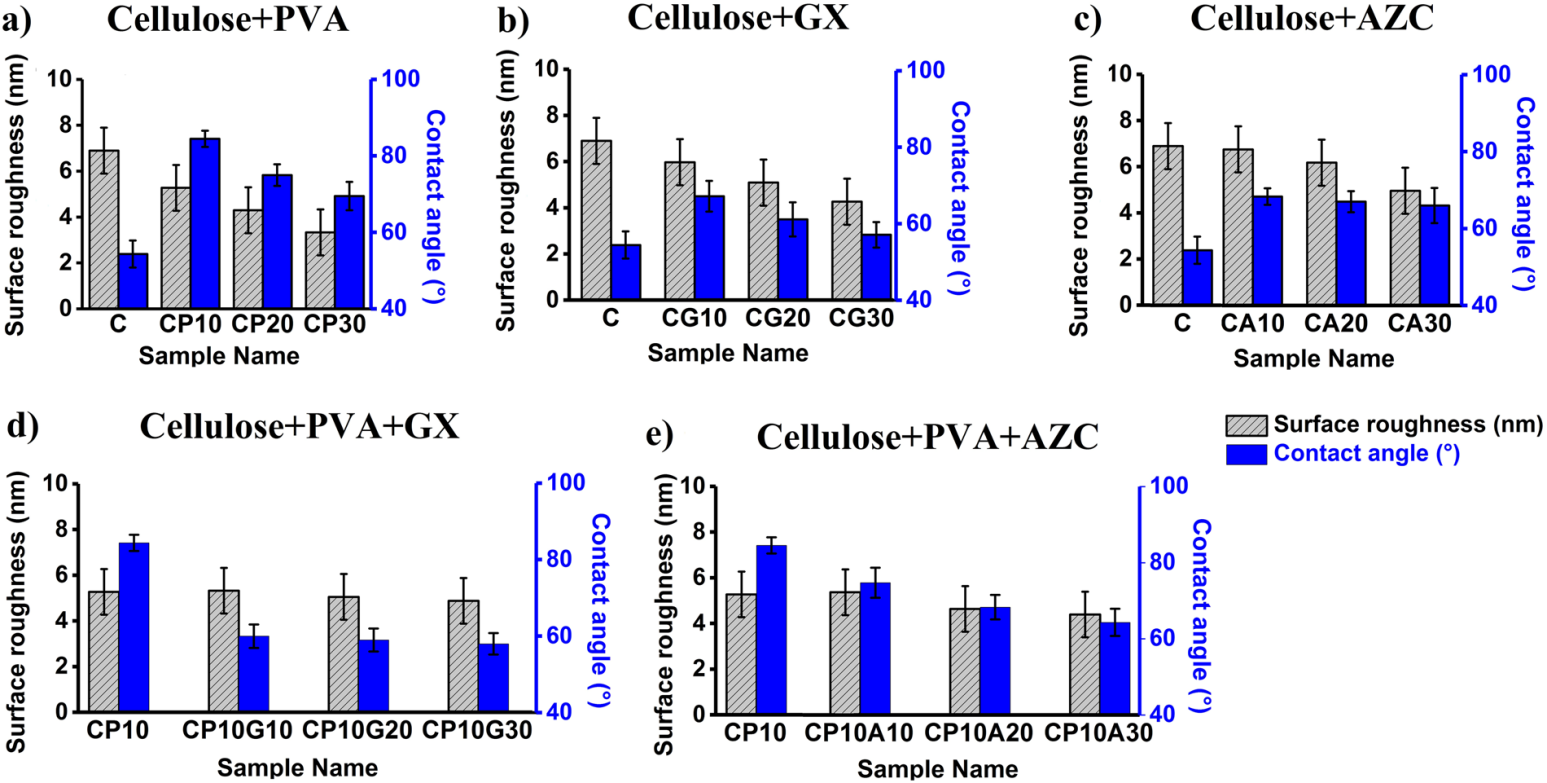
Surface properties of films made of: (**a**) TOCNF and PVA, (**b**) TOCNF and Gx, (**c**) TOCNF and AZC, (**d**) TOCNF, PVA and Gx, (**e**) TOCNF, PVA and AZC.

The water contact angle of the samples as well as the surface roughness values of the film surfaces are presented in Fig. 3. It can be seen that incorporation of PVA improved significantly the contact angle of TCONF films, from 54° (C) to 85° (CP10) (Fig. 3a). In the literature, the WCA of TOCNF films have been reported to be around 50°^5^ and its nanometer surface roughness was attributed to the compactness of the cellulose network, unlike traditional paper (5–10 µm)^1^. The contact angle of a water drop at the air-drop-solid interface depends on the topography and surface energy of the surface^19^ as well as the interactions between the three phases. It can be inferred that the covalent bonding between the carboxyl groups of TOCNF and the hydroxyl groups in PVA, results in smoother surfaces, as observed in the AFM images. However, a lowered WCA of 70° was measured at PVA content of 30%, probably due to the excess unreacted OH that interacts with water.

Incorporation to TOCNF of 10% crosslinkers (Gx or AZC), improved the hydrophobicity but to a limited extent compared to PVA (Fig. 3a–c). The highest addition level of Gx, 30%, reduced the WCA, bringing it back to the value of pristine cellulose while the amount of AZC did not influence the WCA. Interestingly, when Gx was added to CP10, the contact angle was reduced drastically. Compared to Gx, AZC had a reduced impact on the hydrophobicity of CP10. It is proposed that the addition of PVA and crosslinkers mostly decreased the porosity of the samples, making it more resistant to water wetting. A contact angle of around 102° was observed for the PVA film due to a compact structure, strong hydrogen bonding between OH and the presence of few hydroxyl groups on the film surface (not presented in Fig. 3).

### Optical Properties

**Figure 4:**
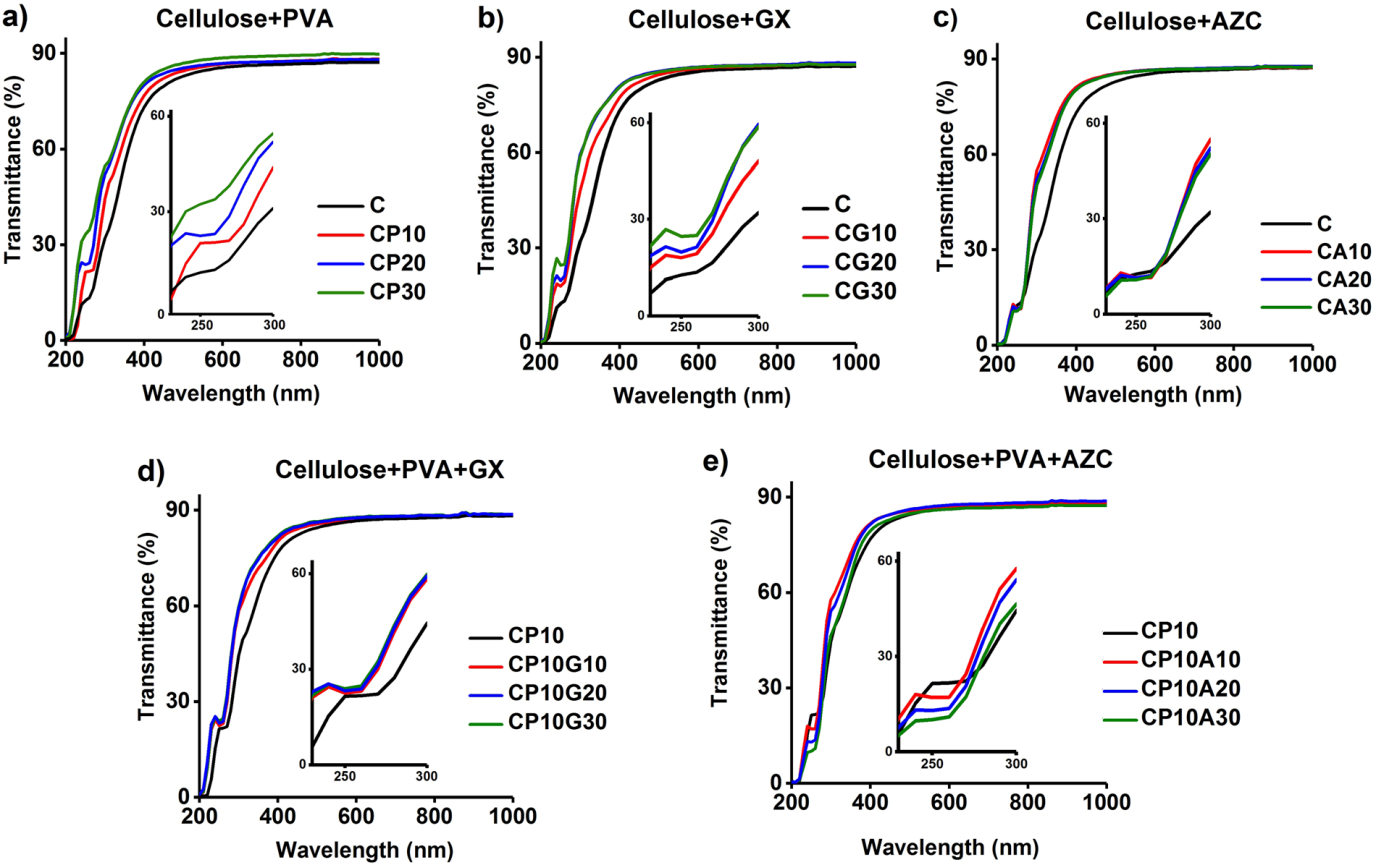
Light transmittance of films made of: (**a**) TOCNF and PVA, (**b**) TOCNF and Gx, (**c**) TOCNF and AZC, (**d**) TOCNF, PVA and Gx, (**e**) TOCNF, PVA and AZC.

Filling the pores and voids between the cellulose network as well as avoiding aggregation lead to reduced films porosity and thus less light scattering and higher transmittance^1^. In this study, films with thickness and density of 40 ± 5 µm and 1.55 ± 0.19 g/m^3^ were obtained, respectively. Figure 4 indicates 84.6–88% transmission of the incident light at 550–1000 nm, which is in good agreement with literature^5^. Similar trends in the infrared region (700–1000 nm) as well as along the visible region (400–700 nm) were observed in the spectra of each sample. The incorporation of the different additives into the cellulose network altered mainly the characteristics of the films in the ultraviolet (UV) region (200–400 nm). An enhanced light transmittance of films in this region is of importance for some UV optoelectronic devices, such as light emitting diodes (LEDs)^20^ and photodiodes^21–23^.

For pure TOCNF film, a high transmittance value (84.6%) was observed at 600 nm and the incorporation of the additives enhanced the transmittance, reaching 88% maximum transmittance for CP30 sample. On the other hand, the shoulder at around 250 nm increased by incorporation of PVA (Fig. 4a) and Gx (Fig. 4b), suggesting the presence of more aldehyde groups at C6 due to the esterification reaction^2^. In contrast, introducing AZC to TOCNF did not influence the shoulder intensity, but enhanced the transmittance at 250–500 nm (Fig. 4c). Interestingly, introducing AZC to TOCNF leads to a lower intensity of the absorbance shoulder compared to the Gx-crosslinked film (Fig. 4b,c). The effect of crosslinkers on the CP10 samples was slightly different compared to that for the pristine cellulose with one additive only. In the former case, Gx did not affect significantly the peak intensity or spectra whereas the peak intensity became slightly lower as AZC was added (Fig. 4d,e).

### Classification and Regression Analysis with the Random Forest Method

**Figure 5:**
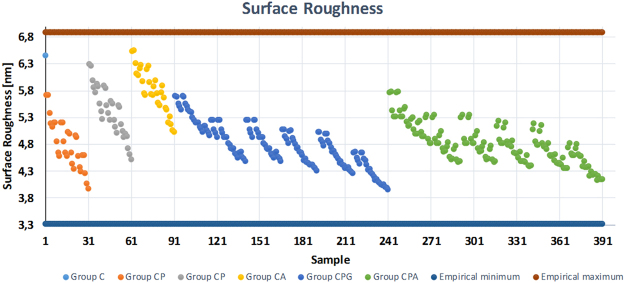
Surface roughness prediction of samples with different cellulose, PVA and crosslinker ratio.

**Figure 6:**
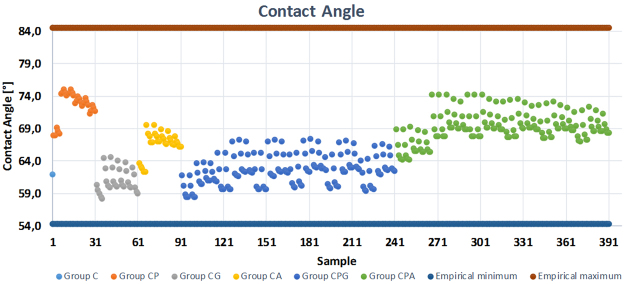
Contact angle prediction of samples with different cellulose, PVA and crosslinker ratio.

**Figure 7:**
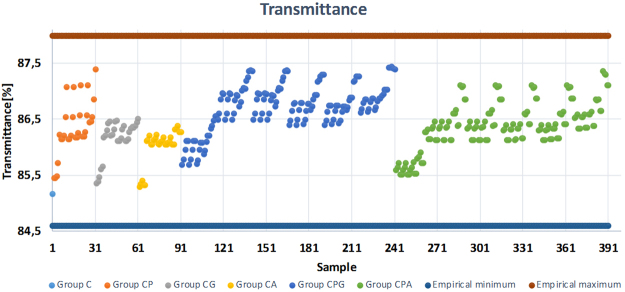
Light transmittance prediction of samples with different cellulose, PVA and crosslinker ratio.

Target physical properties of films are obtained usually after through tedious experimental work and analysis. A computational tool can possibly reduce such explorations by predicting the characteristics of hybrid films. In this study, prediction and regression analyses were carried out with 391 hypothetical inputs (films with different amounts and combination of TOCN, PVA, Gx and AZC) as well as three outputs: surface roughness, contact angle and optical transmittance, as presented in vertical axes of Figs 5–7, respectively. The horizontal axes of these graphs consist of the inputs, which were sorted according to pre-defined rules as explained below (Rule 1 and Rule 2). By analyzing the experimental results and learning from the experimental data, possible substrate outcomes were predicted within the investigated/experimental data range by using the random forest method thus, extrapolation is beyond the scope of this work.

In each case, the experimental minimum and maximum values were used to constrain the predicted outputs. A neat cellulose film was selected as sample 1 and represented in the x-axes of the graphs correspondingly on the left most side. Samples 2–91 were assigned to the two-component systems (CP, CG and CA, in the presentation order along the x-axes) while samples 92–391 were designed as three-component system (CPG and CPA, respectively). Tables 1 and 2 explain the rule of two-component systems (Rule 1) and three-component systems (Rule 2) using CP and CPG categories as examples, respectively. Similarly, the rules can be applied to the rest of the input space.

**Table 1:** Cellulose and PVA content (grams) in samples 2–31 (CP). Here, for CP sample set, Gx = AZC = 0. Rule 1 is also applied to CG and CA films presented in samples 32–61 and samples 62–91, respectively.

Sample	Sector	C	Pva	Rule 1
2–6	1	0.95	0.057–0.285	Input space of CP is divided into six sectors. In each sector, C is constant and it decreases as the number of sector increases. On the other hand, PVA increases from 0.057 to 0.285 in five steps inside a sector and the sector number increases when PVA reaches to 0.285.
7–11	2	0.893	0.057–0.285	
12–16	3	0.836	0.057–0.285	
17–21	4	0.779	0.057–0.285	
22–26	5	0.722	0.057–0.285	
27–31	6	0.665	0.057–0.285

**Table 2:** Cellulose, PVA and Gx content (grams) in samples 92–241 (CPG). Here, for CPG sample set, AZC = 0. Rule 2 is also applied to CPA films presented in samples 242–391.

Sample	Sector	C	PVA	Gx	Rule 2
92–116	1	0.95	0.057–0.285	0.057–0.285	Input space is divided into six main sectors as in Rule 1. Differently, third component (Gx) increases from 0.057 to 0.285 while the other two remains constant through five samples. When Gx reaches to 0.285 PVA increases to the next value and Gx starts from 0.057 again. Sector number increases when PVA reaches to 0.285.
117–141	2	0.893	0.057–0.285	0.057–0.285	
142–166	3	0.836	0.057–0.285	0.057–0.285	
167–191	4	0.779	0.057–0.285	0.057–0.285	
192–216	5	0.722	0.057–0.285	0.057–0.285	
217–241	6	0.665	0.057–0.285	0.057–0.285	

### Accuracy of predictive model

**Figure 8:**
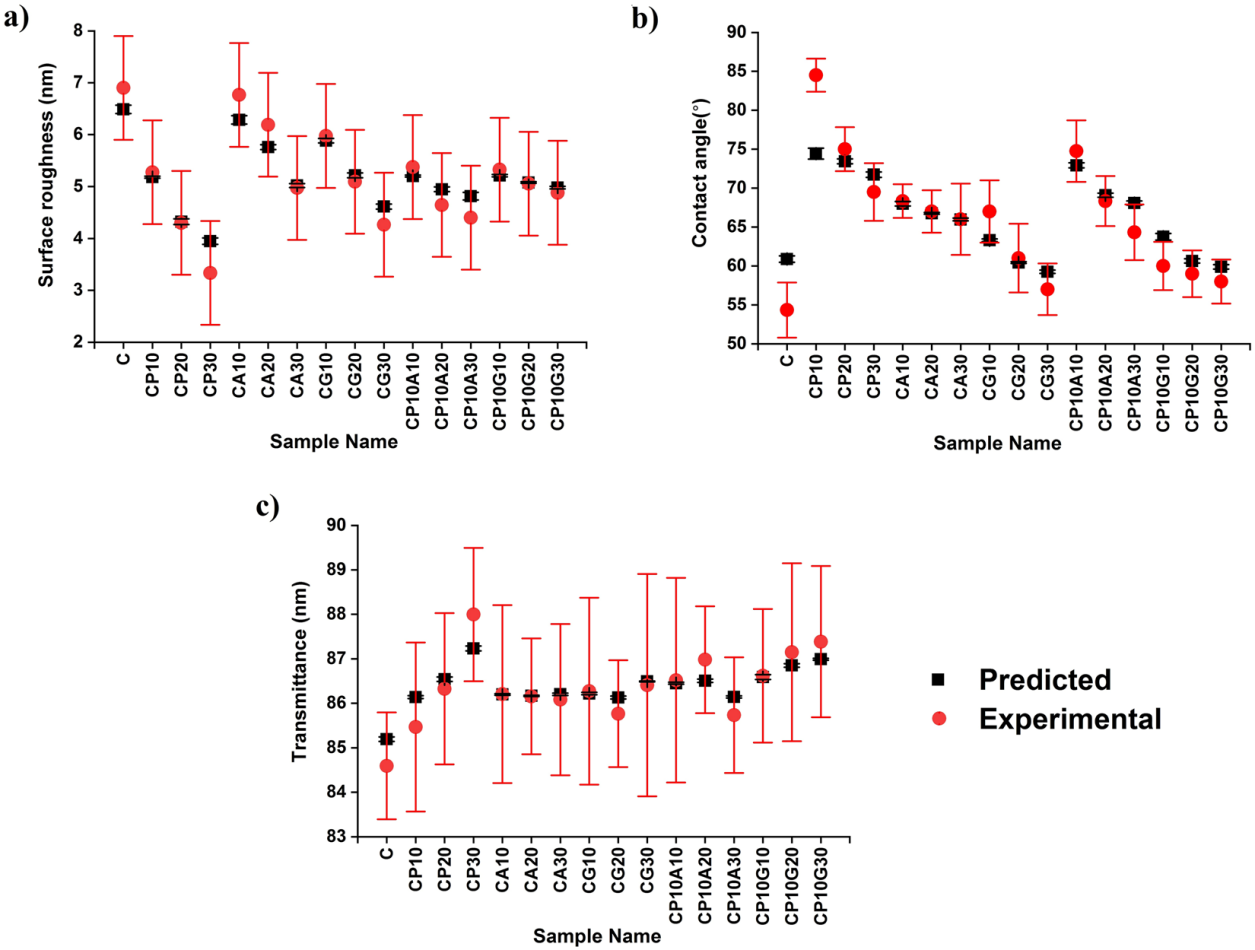
Experimental and predicted data: (**a**) surface roughness, (**b**) contact angle and (**c**) transmittance.

Given the mean absolute percentage error (MAPE) (Equation 2) in Methods section, the measure used for accuracy was calculated as 4.9, 3.9 and 0.3% for surface roughness, contact angle and transmittance outputs, respectively. The accuracy of the prediction suggests that the data training was handled with a relatively small prediction error^24^. On the other hand, it is well known that there is no definition of “precise” prediction in the literature^25^. MAPE of transmittance is almost 10 fold less compared to the other segments of data set with an apparent reason that the constraint boundaries of the data set were quite different (Figs 5–7). Here, the prediction model learns from the experimental results and predicts the corresponding outputs of not only the generated input values but also the existing input values. Therefore, comparing the experimental results with the predicted data can be used as another way to evaluate the accuracy of the prediction (Fig. 8). From Fig. 8, it can be seen that experimental data and predicted data follows similar trends for all three cases with minor differences. Here, the prediction variation of each data set was obtained by running the algorithm five times.

The surface smoothness of a substrate is an indicator of the surface porosity and compactness. According to the experiment data, the roughness of the films varies between 3.3 nm and 6.6 nm. The roughest surface was predicted to be belong to the film with (C, PVA, Gx, AZC) = (0.95, 0, 0, 0.114), respectively. This seems logical because according to the experimental data, pristine cellulose and CA10 samples exhibited almost the same roughness values, which was around 35% more than the average of all samples measured (Fig. 8a). Additionally, 391 samples had an average surface roughness of 4.9 ± 0.5 nm and the roughest surface values were found for the CA samples. On the other hand, both of the three-component systems exhibited statistically almost the same surface roughness regardless of the secondary additive, GX or AZC.

The goniometric study of the surface with a drop determines the wetting capability of the films with the liquid^26^. In this regard, the proposed predictive tools can provide insights about the interactions before an actual application step, for instance, a device integration process. The practical limits of the contact angle studies were between 85° and 54°. The prediction studies indicated that the highest water contact angle (75°) was observed on the films with (C, PVA, Gx, AZC) = (0.893, 0.171, 0, 0) or (C, PVA, Gx, AZC) = (0.836, 0.171, 0, 0). In contrast, the experimental data showed that 85° can be obtained with CP10 having (C, PVA, Gx, AZC) = (0.855, 0.095, 0, 0). Additionally, the lower marginal was found to be 58° predicted to be in the sample with (C, PVA, Gx, AZC) = (0.95, 0, 0.285, 0) whereas the neat cellulose had a contact angle of 54° (Fig. 8b).

The prediction analysis of the transmittance shows that the most transparent (87.5% transmittance) and opaque (85.2% transmittance) films can be obtained using (C, PVA, Gx, AZC) = (0.665, 0.285, 0.171, 0) and (C, PVA, Gx, AZC) = (0.95, 0, 0, 0), respectively (Fig. 8c). Such a prediction is in line with the analysis carried out on the experimental results since the scattering of the cellulose network is reduced by filling the voids with the polymer and Gx, which can promote a network with less voids.

**Figure 9:**
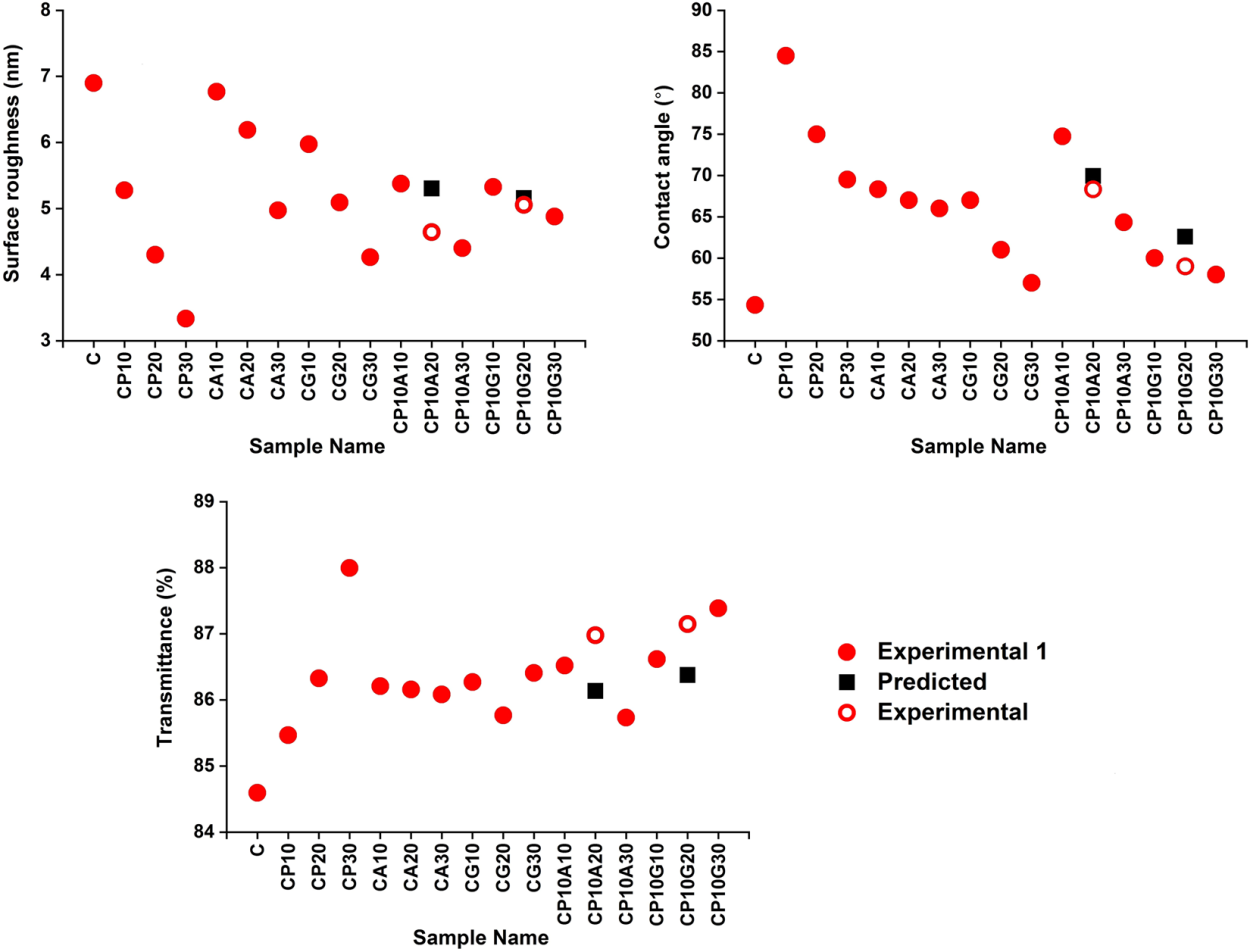
Experimental and predicted data: (**a**) surface roughness, (**b**) contact angle and (**c**) transmittance.

The accuracy of the method was also tested by using fewer inputs. In this case, experimental results of CP10A20 and CP10G20 were excluded and the output prediction was conducted without the inputs, CP10A20 and CP10G20 (“Experiment 1”, in Fig. 9). Thereafter, the predicted values (“Predicted”, in Fig. 9) were compared to the real outputs of CP10A20 and CP10G20 (“Experimental”, in Fig. 9). As a result, surface roughness, contact angle and transmittance of CP10A20 and CP10G20 samples were predicted with 14, 2, 1% and 2, 6, 1% errors (error = 100xdifference between experimental value and predicted value/experimental value) for CP10A20 and CP10G20 samples, respectively. The error in this case was bigger than the previous assessment, albeit staying still in favorable amount^24^. Note that in order to increase experimental precision, the following procedure was followed: (1) All the samples were prepared, characteristics were measured and the experimental data of 16 types of films were obtained. (2) Data of two samples (~13% of the whole data set) was excluded from the training set. (3) Data was trained and the corresponding outputs were predicted.

## Discussions

We demonstrated an example of the convergence of information technology and materials science to achieve innovative developments based on nanocellulose films^27^. Hybrid films made of TEMPO-oxidized nanocellulose fibers (TOCNF), a film-forming polymer (PVA) as well as crosslinkers (glyoxal, Gx and ammonium zirconium carbonate, AZC) were prepared. Optical transmittance and surface properties of the hybrid films were investigated by means of UV-vis spectroscopy and atomic force microscopy and contact angle analysis. Depending on the material composition, given optical transmittance and surface properties were achieved. The highest transmittance was obtained blending 30 wt.% PVA, which resulted in very smooth surfaces while the highest water contact angle was obtained blending only 10 wt.% PVA into the cellulose dispersion. Aiming at the design of custom-made substrates for different applications, a “Random Forest” machine learning method was utilized to train the data by using the experimental results. The surface roughness, contact angle and transmittance properties predicted for the films containing different components were predicted with 4.5, 3.9 and 0.3% MAPE, respectively. The introduction of such a tool for the design of hybrid films can minimize time and costs usually involved in experimental optimization. On the other hand, the prediction performance should be further evaluated with a comparative study with different methods.

## Methods

A fully hydrolyzed, water-dispersible polyvinyl alcohol (PVA, degree of hydrolysis = 99.0–99.8) with molecular weight of ~145,000 (Mowiol 28–99) was obtained from Sigma-Aldrich. Aqueous solutions of glyoxal (40 wt.%) and ammonium zirconium (IV) carbonate were also obtained from Sigma-Aldrich. Commonly, all solutions were used in 0.5% dry weight.

### Film Preparation

2,2,6,6-tetramethylpiperidine-1-oxyl radical (TEMPO) oxidized cellulose nanofibrils were obtained at room temperature and pH 10^2,5^. After the mechanical disintegration in a micro fluidizer (Microfluidics M-110EH-30, Microfluidics Int., USA), 0.5 wt.% aqueous dispersion was obtained after 24 h magnetic stirring followed by centrifugation at 3000 g for 6 min. All samples were prepared from the supernatant dispersion and after mixing with PVA and/or crosslinker 75 minutes before film casting. Films made of pristine TOCNF were used as reference and, referred to as “C”. The effect of PVA on film properties was studied by addition of 10, 20 and 30 wt.% PVA to the TOCNF based on the weight of hybrid films (CP10, CP20 and CP30, respectively). For the crosslinker, 10, 20 and 30 wt.% Gx and AZC were added separately, resulting in films labeled as CG10, CG20, CG30 (for Gx) as well as CA10, CA20 and CA30 (for AZC). The effect of crosslinkers together with PVA was also investigated by applying crosslinkers to 10, 20 and 30 wt.% CP10 solution. The resulting samples are named as CP10G10, CP10G20 and CP10G30 (for Gx) and CP10A10, CP10A20 and CP10A30 (for AZC). Table 3 summarizes the sample compositions and nomenclature. For all three additives, 30% was found to be the approximate upper limit before the dispersion starts to contain bundles after preparing the hybrid dispersion.

**Table 3:** Composition of the films made of TOCNF (C), PVA and crosslinkers (Gx and AZC) at different compositions (dry weight in gram).

Sample Name	C (g)	PVA (g)	Gx (g)	AZC (g)
C	0.95	0	0	0
CP10	0.855	0.095	0	0
CP20	0.76	0.19	0	0
CP30	0.665	0.285	0	0
CG10	0.855	0	0.095	0
CG20	0.76	0	0.19	0
CG30	0.665	0	0.285	0
CA10	0.855	0	0	0.095
CA20	0.76	0	0	0.19
CA30	0.665	0	0	0.285
CP10G10	0.855	0.095	0.0855	0
CP10G20	0.855	0.095	0.171	0
CP10G30	0.855	0.095	0.2565	0
CP10A10	0.855	0.095	0	0.0855
CP10A20	0.855	0.095	0	0.171
CP10A30	0.855	0.095	0	0.2565

The dispersions were casted in polypropylene Petri dishes (13.5 cm diameter) and dried in room condition. A filter paper (Schleicher & Schuell, 240 nm) was kept on the top of the petri dish in order to maintain a uniform vapor pressure during drying, resulting in a uniform film thickness. The average thickness values were calculated by taking five measurement points from each sample (one at the center and four at the edges of the samples).

### Characterizations

The transmittance profiles of the films along 200 nm and 1000 nm wavelength frame were identified with a Perkin Elmer lambda 950 UV-Vis spectrometer. A CAM 200 optical goniometer from KSV instruments with a camera and dispenser system were used to monitor the sessile drop on the surface of the films at 50% relative humidity and 23 °C. The initial contact angle of the drop was calculated in accordance with Young’s equation by embedded image processing program. The surface morphologies of the samples were studied with an atomic force microscope (AFM), Multimode 8, including a NanoScope V controller (Bruker Corporation, Billerica, MA), operating in tapping mode. A NanoScope 8.15 software (Bruker) was used to process obtained images and calculate the surface roughness values. No image processing (except flattening) was performed during this step.

### Random Forest Method

The random forest method, or more specifically Breiman–Cutler ensembles of decision trees, was used as a machine learning technique for classification and regression analysis. This approach is based on decision combination model *g* obtained from a sequence of models *f*. It can be broadly represented as:

1 $${\rm{g}}({x}_{1},\ldots ,{x}_{n})=\sum _{j}{f}_{j}({x}_{1},\ldots ,{x}_{n})$$

This technique uses several models to obtain predictive performance, known as model ensemble, where each *f* is called as a decision tree^28^. It is noteworthy that the models are independently formed through individual data sampling. Thus, the random forest model is efficient for numerical data handling and capturing linear and non-linear interactions between the input and output data^16^. Given that a priori assumption in statistical processes for establishing relationship between input and output parameters, i.e. regression analysis, is necessary, the random forest model is preferred in the current study. In the present study, for regression analysis and predictions, built-in predictor function, Predict[] with “RandomForest” method setting provided in Mathematica technical computing software, is used. The random forest method can deal with various types of variables in one data set^29^. On the other hand, in big data sets, the prediction accuracy drops were reported, therefore, various modifications were proposed^30,31^.

After applying the random forest model as a prediction method, the mean absolute percentage error (MAPE) was calculated from:

2 $${\rm{M}}=\frac{100}{n}\sum _{t=1}^{n}|\frac{{A}_{t}-{F}_{t}}{{A}_{t}}|$$

where, *A*_*t*_ is the actual value and *F*_*t*_ is the forecast value of data set consisting of *n* samples^32^. MAPE was selected due to its simplicity and because of the fact that it applies to data sets that do not contain close to zero or negative values^33^.

### Data availability

The authors declare that the data supporting the findings of this study are available within the article and Mathematica technical computing software is available in https://www.wolfram.com/mathematica/.
